# Material to system-level benchmarking of CMOS-integrated RRAM with ultra-fast switching for low power on-chip learning

**DOI:** 10.1038/s41598-023-42214-x

**Published:** 2023-09-11

**Authors:** Minhaz Abedin, Nanbo Gong, Karsten Beckmann, Maximilian Liehr, Iqbal Saraf, Oscar Van der Straten, Takashi Ando, Nathaniel Cady

**Affiliations:** 1 University at Albany, College of Nanotechnology, Science and Engineering, Albany, NY 12203 USA; 2 IBM Thomas J. Watson Research Center, Yorktown Heights, NY 10598 USA; 3 NY CREATES, Albany, NY 12203 USA; 4 IBM Research, Albany, NY 12203 USA

**Keywords:** Electronic devices, Electrical and electronic engineering

## Abstract

Analog hardware-based training provides a promising solution to developing state-of-the-art power-hungry artificial intelligence models. Non-volatile memory hardware such as resistive random access memory (RRAM) has the potential to provide a low power alternative. The training accuracy of analog hardware depends on RRAM switching properties including the number of discrete conductance states and conductance variability. Furthermore, the overall power consumption of the system inversely correlates with the RRAM devices conductance. To study material dependence of these properties, TaOx and HfOx RRAM devices in one-transistor one-RRAM configuration (1T1R) were fabricated using a custom 65 nm CMOS fabrication process. Analog switching performance was studied with a range of initial forming compliance current (200–500 µA) and analog switching tests with ultra-short pulse width (300 ps) was carried out. We report that by utilizing low current during electroforming and high compliance current during analog switching, a large number of RRAM conductance states can be achieved while maintaining low conductance state. While both TaOx and HfOx could be switched to more than 20 distinct states, TaOx devices exhibited 10× lower conductance, which reduces total power consumption for array-level operations. Furthermore, we adopted an analog, fully in-memory training algorithm for system-level training accuracy benchmarking and showed that implementing TaOx 1T1R cells could yield an accuracy of up to 96.4% compared to 97% for the floating-point arithmetic baseline, while implementing HfOx devices would yield a maximum accuracy of 90.5%. Our experimental work and benchmarking approach paves the path for future materials engineering in analog-AI hardware for a low-power environment training.

## Introduction

In recent years, neural networks have been applied to challenging problems such as image recognition and natural language processing, with the ability to surpass human-level accuracy^1,2^; however, a tremendous amount of power is required to train these models. For example, ChatGPT (which is a version of the GPT-3 model) required 1,287 MWh of power for training^3^. The equivalent CO_2_ emissions for training this model are 552 metric tons, which is around 110 years of an average person’s CO_2_ emission^4^. The computational power requirements to train a neural network (NN) is the result of large amounts of data transfer and weight updates, as well as repeated matrix multiplication operations.

**Figure 1:**
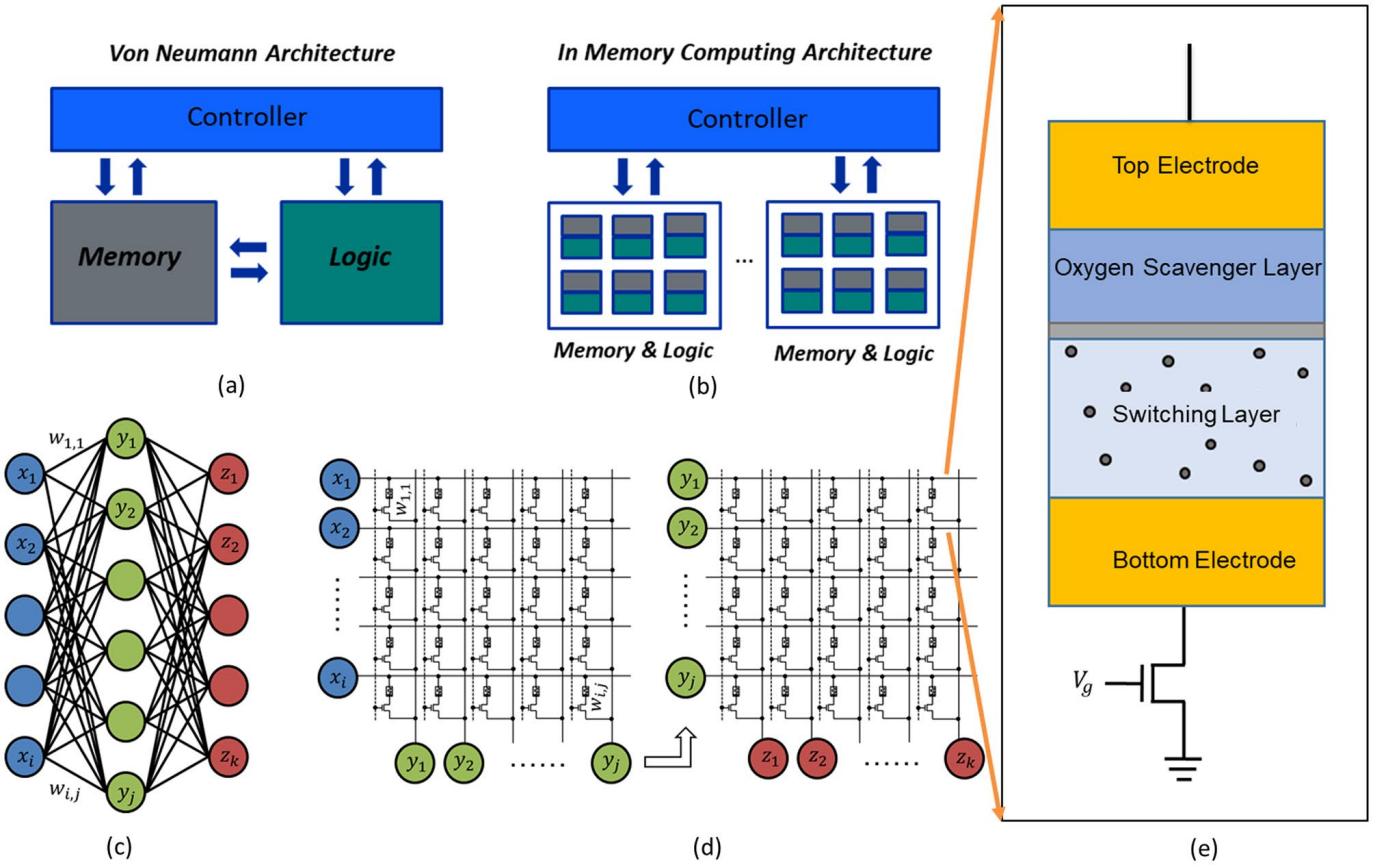
Analog computation of AI workloads is a promising solution to this power-intensive task. RRAM based AI harware accelerators leverage in-memory computation compared to state-of-the-art von Neumann architectures. (**a**) The von Neumann computing architecture consists of physically separated memory and logic units, which are bottlenecked by data transfer (shown by the blue arrow). (**b**) In-memory computation reduces power and latency by computing directly within the memory unit and also utilizes the inherent parallelism of the memory architecture. (**c**) A simple neural network is shown with input layer (blue), hidden layer (green), and output layer (red). (**d**) An example of implementing a RRAM-based memory array for hardware realization of a neural network where each RRAM stores synaptic weight values. Simultaneous input at each row as voltage can result in column current for multiply and accumulate (MAC) operations. It should be noted that MAC operations are a significant contributor towards overall neural network training workload. (**e**) A 1-transistor 1-RRAM (1T1R) unit cell is shown, highlighting a cross-section of the RRAM structure with oxygen vacancies in the switching layer depicted as grey dots.

The conventional von Neumann computing architecture physically separates memory and logic units and has limited parallelism. Leveraging parallelism and repeated multiplication operations, GPUs increase the training throughput significantly^5^. As data transfer between memory and logic units introduce significant power consumption and latency, in-memory computation holds further opportunities for power and efficiency improvements^6^ (Fig. 1a,b). Additional power and latency reduction can be achieved by analog computation of multiplication operations. Studies have predicted that approximately 100 to 1000 times more efficient neural network training is possible on an analog, in-memory processing unit, as compared to state-of-the-art GPU computing^7^.

**Figure 2:**
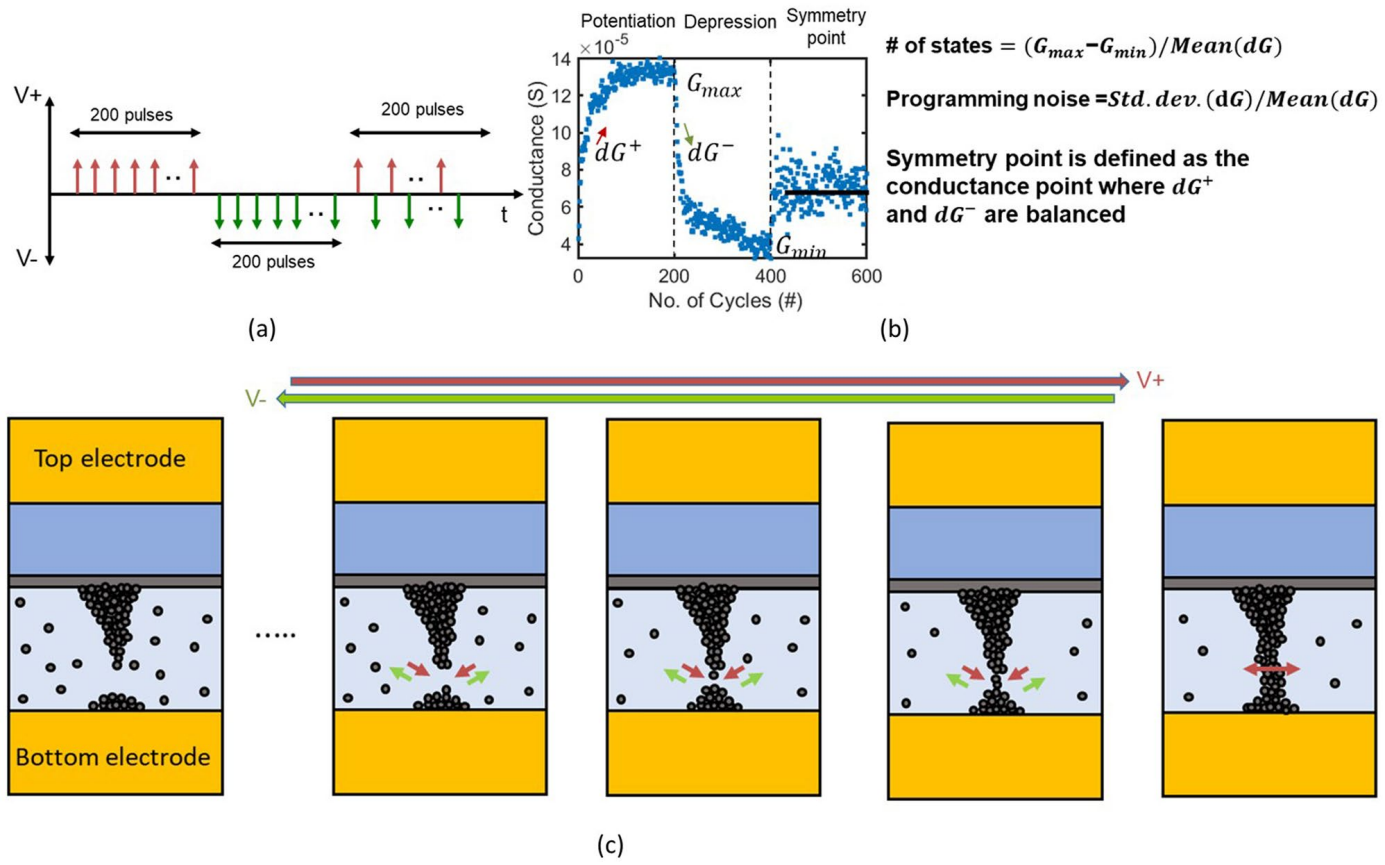
(**a**, **b**) Electrical pulses and polarities required for analog switching. Short-duration positive pulses increase the conductance (or weight) of the device whereas negative pulses reduce RRAM device conductance. The repeated alternation between positive and negative pulses results in the device achieving the so-called symmetry point, where successive positive and negative pulses do not significantly alter device conductance. Number of states and programming noise definitions are shown on the right. (**c**) Schematic of the physical changes occurring to the conductive filament of a RRAM device during positive and negative pulses for analog switching using hour-glass model^13,14^. Positive pulses across the device grow the filament towards the bottom electrode, hence reducing the oxide gap in between. This increases the overall device conductance, whereas the opposite behavior occurs during negative pulses across the device, decreasing the device conductance.

Resistive Random Access Memory (RRAM) based analog accelerators have shown promising results for low-power and high-speed neural network training^5,8–12^. RRAM-based memory is non-volatile in nature with analog data storage capabilities (Fig. 1e). RRAM are typically configured as a metal-insulator-metal (MIM) structure consisting of a top and bottom electrode with a switching layer in between. These devices store data as measurable conductance states, controlled by the position and relocation of ions within a conductive filament (Fig. 2). In oxygen vacancy based switching devices, a conductive filament is formed within the switching layer, connecting the bottom and top electrode. The filament consists of an accumulated column of oxygen vacancies^13^. An increase of the vacancy concentration (typically near the bottom electrode) will increase the conductance of the filament and thus change the conductance state of the RRAM. Likewise, under reverse bias, the concentration of oxygen vacancies can be reduced, yielding lower conductance. The conductive filament can be modified gradually by consecutive short pulses, making RRAM devices a viable option for synapse-like weight storage^14^ (Fig. 2). In addition, RRAM arrays can be used to perform analog vector matrix multiplications or multiply–accumulate (MAC) operations by coding the input vector into a range of voltages and the matrix weights into conductance states within the array. The resulting output current vector (y_*n*_) contains the encoded result of the operation (Figure 1 (c,d)). Analog matrix multiplication results can be achieved within a single cycle and therefore have a computational complexity of *O*(*n*) compared to *O*(*n*^3^) with conventional logic.

The most important performance metrics of the RRAM-based hardware accelerators are training accuracy and system power consumption. Training accuracy depends on the device switching behavior, such as the linearity, asymmetry during the process of potentiation or depression (increasing or decreasing the synaptic weight)^15,16^. Overall training accuracy also depends on the number of conductance states (defined as the conductance range divided by mean conductance change at the symmetry point) and programming noise (standard deviation of the change of conductance divided by mean change of conductance at the symmetry point) (Fig. 2b)^17,18^. Generic neural network algorithms (e.g Stochastic Gradient Decent (SGD)) depend on ideal device weight updates (linear, symmetric and large number of states), and deviation from linear weight update results in a decreased training accuracy^19,20^. Most reported analog device behaviors with RRAM devices, however, are non-ideal with a non-linear asymmetric weight update, low number of states, and a relatively large noise level^8,21^. Hence, there is a need for a training algorithm that can handle the imperfect behavior of the RRAM devices and still provide high training accuracy.

In this work we adopted a analog fully in-memory training algorithm named Tiki-Taka v2 (TTv2) that embraces practical non-ideal device switching behavior without compromising performance^17,22^. TTv2 results in higher accuracy compared to the generic SGD algorithm with a comparatively lower number of states, high non-linearity, and variations^17^. This algorithm relies on three RRAM unit cells, one RRAM for analog conductance/weight update and the second RRAM to store the “symmetry point” as a reference point between positive and negative weights, one RRAM for gradient accumulation around symmetry point^23^. This RRAM conductance “symmetry point” can be achieved by juxtaposing positive and negative pulses^23,24^ (Fig. 2). Other device properties like conductance values significantly influence overall system power consumption while reducing IR drop^10^. The device switching linearity/non-linearity and conductance of the device are significantly influenced by the material stack of the RRAM device^15,16^. As a result, a RRAM-based accelerator’s overall efficacy depends largely on its material selection. For high volume manufacturability, conventional complementary metal oxide semiconductor (CMOS) fabrication compatibility of the RRAM material stack is one of the most important factors^13^. Due to proven CMOS compatibility, available material deposition/process tools in the foundry, HfO_x_ and TaO_x_-based RRAM devices have had the highest research interest compared to other stacks^25^. In this work, we focus on benchmarking analog performance such as the number of states, dynamic range, and programming noise of HfO_x_ and TaO_x_ RRAM devices, both fabricated on 65nm CMOS technology process node. Furthermore, we benchmarked system-level training accuracy from both HfO_x_ and TaO_x_ device switching behaviors leveraging the TTv2 algorithm.

In this work, RRAM devices with different switching layers (HfO_x_ and TaO_x_) were fabricated using a standard CMOS process technology (65 nm) for benchmark comparison of their potential for analog performance and utility in neural network training and accelerator approaches. Analog switching experiments with pulse length as short as 300 ps demonstrated the ability to achieve 35 analog states with TaO_x_ versus 29 states with HfO_x_, with TaO_x_ having lower off-state conductance, which is amenable to low power operation. We report the impact of pulse amplitudes on symmetry point convergence was systematically studied for the first time. Finally, we adopted an analog fully in-memory training algorithm (TTv2) for system-level benchmarking of neural network training and accuracy, and report on the baseline RRAM device switching data, fitting, and hyperparameter optimization to yield up to 96.4% accuracy versus a floating point peak accuracy of 97%.

## Results

### Fabricated RRAM device stack

**Figure 3:**
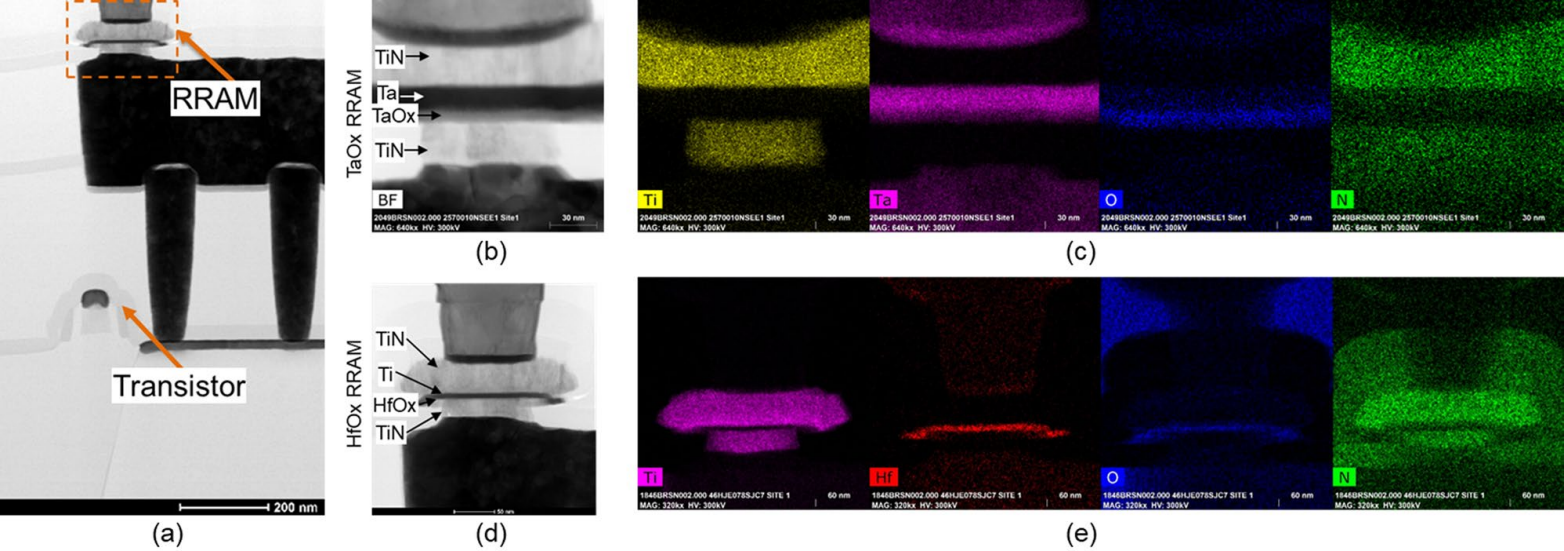
(**a**) Brightfield TEM image of CMOS-integrated RRAM cell in 1-transistor 1-RRAM (1T1R) configuration. Zoomed-in brightfield images of the TaO_x_ (**b**) and HfO_x_ (**d**) based RRAM devices shown on the left. Energy-dispersive X-ray spectroscopy (EDS) images showing the elements Ti, Ta, Hf, O, and N for the TaO_x_ (**c**) and HfO_x_ (**e**) devices shown on the right.

We developed a monolithic integration scheme for embedded RRAM in a 65 nm CMOS process technology based on a 300mm wafer-scale fabrication platform^26^. The RRAM devices were integrated above high voltage I/O FETs (V_*DD*_ = 3.3 V) enabling excellent current control with significantly reduced parasitic capacitance and excellent current compliance control compared to off-chip solutions^27^. RRAM devices were integrated between metal 1 (M1) and metal 2 (M2) interconnect layers in the back end of the line on top of a custom TiN bottom electrode layer (V0). A custom dual-damascene approach (within back end of the line (BEOL) compatible thermal budget of 430 °C) was developed for the via 1 (V1) and M2 enabling the simultaneous connection of V1 to the top of the RRAM and M1. Both HfO_x_ and TaO_x_ devices have the same electrode size and are fabricated using the same mask set. The lateral dimension of the devices is defined by the bottom electrode (with target dimensions of 120 nm x 120 nm). RRAM devices consist of four layers, starting with the separately structured TiN V0 bottom electrode (Fig. 1e). The metal oxide switching layer, HfO_x_ (6.3 nm) or TaO_x_ (7 nm), was followed by either Ti (6 nm) or Ta (12 nm) oxygen scavenger layer, respectively, and with a TiN (40 nm) top and TiN bottom (20 nm) electrode. The oxygen scavenging layer creates an oxygen vacancy gradient and increases the ion mobility within the switching layer^28^. Both HfOx and TaOx device stacks were optimized to achieve forming voltage compatible with the high voltage IO transistor (3.3 V) offered by 65 nm technology node (Supplementary Material). Figure 3 shows a high-resolution STEM image from an aberration-corrected Titan S/TEM including energy-dispersive X-ray (EDX) spectroscopy imaging of the HfO_x_ and TaO_x_-based RRAM devices.

#### Analog switching for fully in-memory training

**Figure 4:**
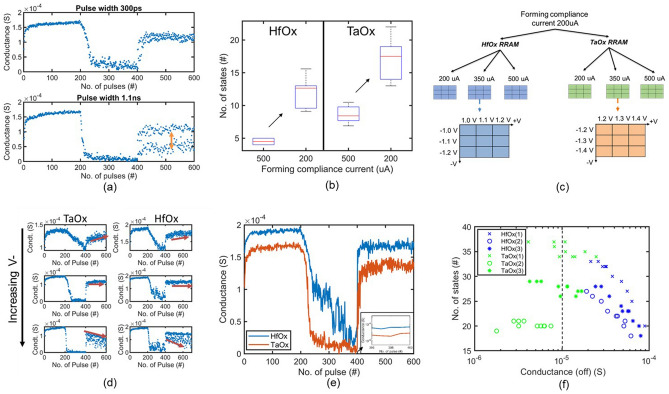
(**a**) Pulse width experiments show the device can switch with ultra-fast 300 ps pulse width while achieving symmetry point convergence. (**b**) Different forming compliance currents for both HfO_x_ and TaO_x_ RRAM devices and the resultant number of states. When formed at lower compliance current for both HfO_x_ and TaO_x_ devices the number of states increases. (**c**) The approach for adjusting the compliance current during initial electroforming and subsequent set and reset voltage ranges explored for both HfO_x_ and TaO_x_ devices. (**d**) Symmetry point shift with pulse condition. For both HfO_x_ and TaO_x_ devices it shows lower negative pulse amplitude can lead to symmetry point shift up whereas high negative voltage can result in symmetry point shift down. (**e**) Analog switching conditions with a high number of states and low off-conductance. Inset shows using a semi-log plot that TaO_x_ device conductance is *∼*10× lower. (**f**) No. of states VS off conductance showing, both HfOX and TaO_x_ devices show 15 states but only TaO_x_ devices can achieve over 10 µ*S* conductance.

The RRAM devices in this work store data in resistive states via anion movement within a conductive filament (Fig. 2). The filament is formed via a controlled dielectric breakdown creating an accumulation of oxygen vacancies within the metal oxide switching layer that is bounded between the top and bottom electrode. A change in oxygen vacancy concentration allows for the modulation of the conductance and enables an analog control. An initial electroforming event is executed via a positive voltage pulse to the top electrode and subsequent negative and positive voltage pulses will reset and set the device. The reset and set process refers to the decrease and increase in conductance and corresponds to the decrease and increase in the oxygen vacancy concentration of the filament, respectively. Two methods are deployed to modulate the electrical stimulus are a pulse width and amplitude change of the applied voltage (Fig. 2). For example, typically application of large pulse width signals (1–100 µs) causes an abrupt change in conductance within a single pulse, resulting in only two states or binary/digital data storage; however, consecutive pulses (e.g. 200 pulses) with shorter pulse widths (typically less than 100 ns) result in a gradual change in the conductance. These conductance/weight updates can then be leveraged to emulate a synaptic behavior for on-chip learning. Gradual increase of the device conductance, called potentiation, can be consecutive positive voltage pulses across the devices. Alternatively, consecutive negative voltage pulses decrease the device’s conductance, also known as depression. By applying many alternating positive and negative pulses in sequence, the resulting RRAM conductance can converge to a symmetry point (as defined above). Analog switching behavior, inclusive of potentiation, depression and symmetry point, is dependent on applied pulse width. Figure 4a shows that with longer pulse width (1.1 ns) the symmetry point fails to converge. Both pulse width conditions were performed on a device formed with 200 *µ*A peak compliance current and pulse amplitudes of +1.0 V, − 1.2 V applied while allowing maximum 500 *µ*A during switching.

#### Dependence on RRAM forming conditions

Low-power analog computation requires the RRAM conductance to be low, as this results in reduced current and overall power consumption during switching and read operations. Our goal with analog switching experiments was to achieve a low off-state conductance of the devices while maintaining analog performance, including the number of states and programming noise. For these experiments, RRAM devices were formed with pulse amplitude of 4.0 V and pulse width of 10 ms for two different compliance currents 200 µA and 500 µA (controlled by the integrated control transistor). Each set of devices was then subjected to analog switching conditions with + 1.2 V, − 1.2 V and + 1.3 V, − 1.3 V for HfO_x_ and TaO_x_ devices, respectively. In both cases, the current compliance was set to 350 µA. The results show a significant increase in the number of achievable conductance states for both HfO_x_ and TaO_x_ devices if a lower compliance current is used during the forming event (Fig. 4b).

For TaO_x_ devices, the average number of states increased from *∼*8 to *∼*18. In the case of HfO_x_ devices, the number of states increased from *∼*5 to *∼*13. For HfO_x_ devices, the conductance slightly decreased from 108 to 98 µS for 500 to 200 µA compliance current during forming. In the case of TaO_x_, however, a greater reduction in conductance of 31–17 µS was observed for the off state.

#### RRAM analog switching

Due to the resulting high number of states and low conductance in the HRS, a compliance current of 200 µA was used during the RRAM electroforming event, prior to pulse amplitude experiments. HfO_x_ devices yielded the best analog switching performance for set voltages of 1, 1.1 and 1.2 V and reset voltages of − 1, − 1.1 and − 1.2 V. Results were similar for TaO_x_, where the range was 1.2–1.4 V and − 1.2 to − 1.4 V for the set and reset operation, respectively. Preliminary experiments showed that the voltage range for analog switching of HfO_x_ and TaO_x_ devices required different voltage ranges. For the same pulse width, TaO_x_ devices required a 0.2–0.3 V higher voltage, agreeing with previously published results^29^. To enable a fair comparison of the analog switching performance, we performed analog switching experiments with the electrical switching conditions that yield the largest number of states as well as the lowest conductance (off state). It should be noted that analog switching is only possible within a well-defined voltage range. Outside of this range, low voltage pulsing will not change the conductance, while large voltage pulses outside of the range will transition the device into binary switching mode, with a small memory window. During all switching conditions described above the pulse width was kept constant at 300 ps with a 70 ps rise and fall time.

To study the effect of peak current during analog switching, a pulse amplitude experiment was performed at three different compliance currents, as controlled by the integrated transistor: 200, 350 and 500 µA (Fig. 4c) for both HfO_x_ and TaO_x_. For each current level, nine pulse amplitude switching conditions were applied. Table 1 shows the converged symmetry points and the comparatively large number of states for each case. Additionally, the larger compliance currents (350 and 500 µA) enable the optimal combination of the lowest conductance and highest number of states. It should be noted that even with the same forming process and switching compliance current, the number of conductance states and the off-state conductance depends on different switching pulse amplitudes (Table 1). These conditions also maintain programming noise of less than 1. For TaO_x_ devices, the analog switching compliance current of 500 µA and pulse amplitudes of +1.4 and − 1.3 V yielded the greatest number of states (35) with off state conductance of 9.8 µS. The highest off state conductance of 3.8 µS from TaO_x_ devices results from 500 µA and pulse amplitude of +1.3 V and -1.3 V for set and reset, respectively, with a lower number of states (20). The highest number of states (29) and off conductance of 37 *µ*S for HfO_x_ is a result of a 350 µA compliance current and a set / reset voltage of +1.2 V and -1.1 V, respectively. Based on these measurements, TaO_x_ devices exhibit around one tenth of the off-state conductance compared to HfO_x_ devices. TaO_x_ devices also have a marginally higher number of states (35) compared to HfO_x_ devices (29).

**Table 1:** Experimental results for HfO_x_ and TaO_x_ devices.

Material	CC (µA)	Pulse Amp. (V)		Number of states			P. noise	Conductance (µS)			Accuracy (%)
		V+	V−	Avg	SD	Max		Off	On	D.R	
HfO_x_	200	1.2	1.0	12	0.87	14	0.75	76.9	143	1.86	29.4
		1.2	1.1	12	1	14	0.73	32.3	143	4.43	82.3
	350	1.1	1.1	23	3	28	0.72	45.5	200	4.2	87.4
		1.2	1.1	**29**	4	36	0.73	37.0	200	5.4	**89.7**
	500	1.1	1.1	20	2.8	23	0.68	100	213	2.1	**90.5**
TaO_x_	200	1.4	1.3	11	1.2	14	0.68	10.9	100	9.2	92.9
	350	1.3	1.3	18	2.98	23	0.73	17.0	128	7.8	89.1
		1.4	1.3	27	1.1	29	0.81	8.5	57.5	6.8	84.1
	500	1.3	1.3	20	0.6	21	0.74	**3.82**	167	**43.7**	90.7
		1.4	1.3	**35**	1.2	37	0.75	9.8	179	18.2	**96.4**

Significant values are in bold.

#### Benchmarking of hafnium oxide and tantalum oxide RRAM performance

To compare HfO_x_ to TaO_x_ devices, we followed two main steps: (1) determination of symmetry point convergence, and (2) elucidation of ideal switching conditions based upon the number of states, programming noise, and off state conductance. To implement the TTv2 algorithm with RRAM based hardware, analog switching must achieve a stable symmetry point. If an electrical switching condition does not yield symmetry point convergence, this electrical switching condition cannot be used for hardware implementation into the TTv2 algorithm, regardless of its number of states and off conductance. A converged symmetry point can be defined as a device conductance state that shows a stable minimum conductance change around a mean conductance value, upon the application of consecutive positive and negative voltage pulses. An non-convergent (divergent) symmetry point can be defined as a state where the mean conductance change of the device is increasing, or decreasing. It can also include a symmetry point separation. As an example, Fig. 4 shows that for same positive pulse voltage conditions, the symmetry point tends to shift up if the negative pulse amplitudes are comparatively lower. Further, the symmetry point tends to shift downwards for comparatively larger negative pulses. For each forming and switching compliance current condition, different optimal switching conditions are required to achieve a stable symmetry point.

As previously discussed, the number of analog states in the RRAM device can be calculated by dividing the total con- ductance range by the mean absolute value of the conductance change from the symmetry region as *Number of states* = *G*_*range*_*/mean*(*dG*). The conductance range, *G*_*range*_ calculated from *G*_min_ from the depression cycle and *G*_max_ from the potentiation cycle. To avoid misleading results due to the inherent conductance variation for RRAM devices, an average of the last ten conductance values during potentiation and depression was taken as *G*_max_ and *G*_min_, respectively (Fig. 2b). *dG* is the mean conductance change which can be calculated from the conductance change from one pulse to the next. Another key metric, programming noise, can be defined as the deviation of each conductance state from its predicted conductance value. Lower programming noise results in more accurate AI training, and for TTv2 programming noise is that its required to be lower than 1.

### Analog fully in-memory training accuracy

**Figure 5:**
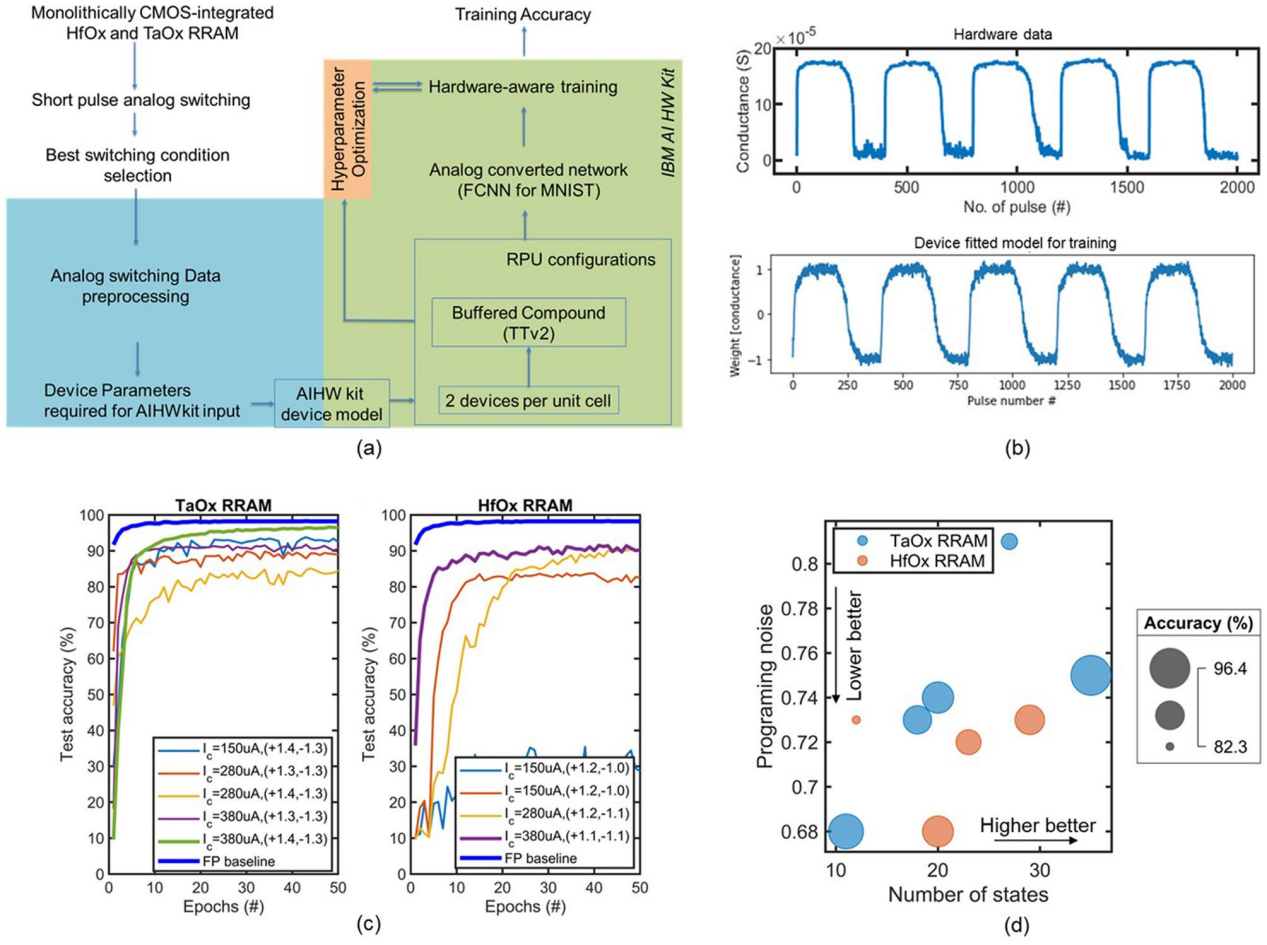
(**a**) Diagram showing the complete workflow from device fabrication, to analog switching experiments, to optimal switching condition selection, to device model fitting, hyperparameter optimization, and finally to analog fully in-memory training. (**b**) Example of hardware data and fitted model for device aware-training. (**c**) Example of hyperparameter optimization. Here the goal is to extract parameter values that yield the highest accuracy without over or under-fitting. (**d**) Accuracy plots for both TaO_x_ and HfO_x_ based analog switching conditions. TaO_x_ based device switching (500 µA + 1.4 V −1.3 V) showing close to floating point baseline accuracy.

Training simulations were performed with a single learning rate, and fast learning rate (AIHWKit parameter), and batch size. In this approach, if the hyperparameters are not optimized, the training accuracy can be misleading. Thus, each analog switching behavior requires its own optimized hyperparameters for training (Fig. 5a). Simulations yielded a baseline floating point accuracy of 97%. Training based on TaO_x_ device metrics yielded close to the floating point-baseline accuracy of 96.4%, whereas training based on HfO_x_ device metrics did not exceed an accuracy of 91% (Figure 5c). The highest training accuracy resulted from TaO_x_ device metrics based on switching with a compliance current of 500 µA and with set and reset voltage of +1.4 V and − 1.3 V, respectively (which enabled 35 conductance states). HfO_x_ devices exhibited 20 and 29 states for 500 µA and 350 µA, respectively with set/reset voltages of + 1.1/− 1.1 V and + 1.2/− 1.1 V, respectively. From the results, it is clear that training accuracy improves with a higher number of conductance states but decreases with higher programming noise. As a result, cases with a reduced number of conductance states with lower programming noise, result in considerable training accuracy (Fig. 5d). For example, HfO_x_ RRAM with switching compliance current = 500 µA where the number of states is 20 and programming noise 0.68 has slightly higher accuracy (90.5%) than 350 µA +1.2 V − 1.1 V condition with number of states 29 and programming noise of 0.73 (accuracy 89.7%) (Table 1). Similarly for TaO_x_ the 350 µA +1.4 V − 1.3 V switching condition (27 states and a programming noise of 0.81) results in lower accuracy than the alternative switching conditions of 350 µA + 1.3 V − 1.3 V (17 states and 0.73 programming noise) (Table 1).

## Discussion

In this work we report on an approach to achieve a high number of states in RRAM-based synaptic devices with corresponding low off state conductance, by first using a reduced compliance current during the forming process and then higher compliance current during subsequent switching events. Previous reports suggest that a minimum compliance current is required to enable RRAM device switching^27^. The authors hypothesized that RRAM devices require a minimum current to assist with oxygen vacancy rearrangement, as a key driver for adjusting device conductance. We extend this hypothesis to explain the low off-state conductance. After forming at a low compliance current, a higher current is required during switching to assist with oxygen vacancy movement. Higher vacancy movement can result in a reduced concentration of vacancies at the end of the filament during the negative pulse cycle. In turn, this can result in lower conductance. Similarly low compliance current would result in lower vacancy movement as a result, higher off-conductance. The change in conductance during negative pulse cycling is affected by the prior forming or set condition that was used, which determines filament size and composition.

Previous reports using analytical simulations^23,30^ assumed that symmetry points can be achieved at any location and with any number of states. Our experiments using fully CMOS-integrated devices show that a stable symmetry point convergence can not be achieved at any indiscriminate location. We observed that symmetry point stability is largely dependent on the amplitude of the positive and negative voltage pulses during the switching (Fig. 4). The symmetry point can shift upward or downward, due to an imbalance of positive and negative voltage pulses. A large positive pulse with a smaller corresponding negative pulse tends to shift the symmetry point upward towards higher conductance. On the other hand, when the negative voltage pulse amplitude is larger, as compared to the positive pulse, the symmetry point tends to shift downwards. For our RRAM devices, symmetry point separation tends to occur when positive and negative voltage pulses are larger e.g. at + 1.5 V − 1.5 V.

As fabricated in this study, CMOS-integrated TaO_x_ RRAM devices exhibited a significantly lower conductance compared to HfO_x_ RRAM devices that were integrated with an identical configuration (1T1R). Conductance of RRAM devices can be attributed to the effect of the different metal-insulator interface, the effect of oxygen scavenging layer, vacancy concentration of the switching layer and bulk oxide conductance. It has been suggested in previous publications that TaO_x_ and HfO_x_ have different defect migration energy for same oxygen scavenging layer metal which can also lead to different conductance values^13,29^. Experimental results reported previously showed strong dependence on the choice of oxygen exchange layer (OEL) for the RRAM stack and on and off conductance of the RRAM device^28^. This suggests further hardware and modeling experiments are required for further understanding and optimization of each material RRAM device stack.

## Conclusion

HfO_x_ and TaO_x_ RRAM devices have great potential as analog switching devices for the implementation of customized AI hardware. We demonstrated that ultrafast (300 ps) switching can not only speed-up the conductance update duration but also benefit analog switching performance. We report on an approach to obtain a large number of conductance states while maintaining low off state conductance by using low compliance current during initial device electroforming, followed by high compliance current switching. We report that TaO_x_ devices have a 10x lower conductance compared to HfO_x_ devices when integrated into 1T1R cells using an identical processing approach. As a result, this makes TaO_x_ devices more suitable for large resistive array-based accelerators. Additionally, we adopted a device-aware training approach with hyperparameter optimization for each switching condition for system-level benchmarking. From this benchmarking approach, we demonstrated that TaO_x_ device performance metrics can yield a higher system-level accuracy of 96.4% as compared to 90.5% for HfO_x_ devices, with accuracy approaching the floating point baseline of 97% accuracy.

## Methods

### Testing

The high-frequency characterization system consists of the pulse generator, power-splitter, oscilloscope, high-frequency compatible probes, and 50 Ω terminator for impedance matching network. A BNC (Berkeley Nucleonics Corporation) Model 765 pulse generator with programmable pulse width down to 300 ps, 70 ps rise/fall times. It was used to generate the forming, switching, and read pulses for RRAM both forming and analog switching experiments. The output of the RF pulse generator fed to the power splitter in two channels (i) 1 channel to the oscilloscope (LeCroy Wavepro 740Zi) to observe the input signal (ii) the second channel to the top electrode of the RRAM device. 50 Ω terminator resistor at the probe ends to minimize the reflected signal and power loss. Another channel of the oscilloscope used to monitor the current through the 1T1R cell. The gate voltages of the integrated transistor were applied using Keysight B1500 parametric semiconductor analyzer as constant DC voltage for a pulse cycle.

### Analog fully in-memory training method

To benchmark the different RRAM devices, we took the analog switching behavior from the devices and feed to an analog fully in-memory training framework. IBM Analog Hardware Acceleration Kit (IBM AIHWkit) is an open-source Python toolkit that allows exploring practical device-aware training^31^. The experimental data first normalized to *W*_max_ and *W*_min_. To support the Wmin and Wmax range, the conductance values first clipped to *G*_min_ and *G*_max_. Specially during depression cycle, it can have large variability. To avoid the random low/high conductivity to be considered as the *G*_min_ and *G*_max_, average of last ten cycles were taken as *G*_min_ and *G*_max_. Then the experimental conductance values were clipped to *G*_min_ and *G*_max_. Then the conductance values were converted to weight values by normalizing to − 1 to + 1 range. First, a generic set of values with the translated number of states^32^ is used to generate a switching behavior. Then following an iterative method of changing the values of piece-wise linear model, to match the potentiation and depression shape while keeping the same symmetry point location and variation (Fig. 5b). The best switching behaviors that show high number of states as well as Roff from both HfO_x_ and TaO_x_ devices, are modeled one at a time.

The modeled AIHWkit device behavior is then fed into the framework for analog fully in-memory training. For analog fully in-memory training, we have used the TTv2 training algorithm which is less susceptible to non-linear weight behavior compared to generic training SGD-only algorithms. TTv2 training algorithm takes advantages of the symmetry point as a reference for up and down weight updates. By zero point shifting of the weights, the algorithm reaches higher accuracy compared to SGD-only algorithms. We used a 3-layer network to train hardware aware training for MNIST handwritten digit classification. For simplicity and repeated benchmarking purposes we used 3 layers fully connected (input, hidden, output) network. We trained floating point training as a baseline to compare analog device-aware training. Hyperparameter optimization of training each device switching was done using Weights & Biases^33^. Each analog switching behavior requires specific set of learning rate, batch size, fast learning rate (a framework-specific parameter for TTv2 unit cell compound). Without these hyperparameters optimized the, it risks over/underfitting. As a result, for each analog device switching behavior requires needed to be run through optimization for specific batch size, learning rate, fast learning. Bayesian optimization experiments were run for each switching behavior with learning rate, fast learning rate ranging from 10 to 0.00001, batch size from 8 to 216. Bayesian search learned from the resultant loss function from previously run parameters (learning rates, batch size) and next set of generated parameters were generated following the trend. These experiments were run by integrating AIHWkit analog training with W&B (weights and biases) optimization add-on.

### Supplementary Information

Supplementary Information.

## Data Availability

The datasets generated during and/or analyzed during the current study are available from the corresponding author upon reasonable request.
