# Supplementary material for: Material to system-level benchmarking of CMOS-integrated RRAM with ultra-fast switching for low power on-chip learning
Source: Sci Rep. 2023 Sep 11;13:14963. doi: 10.1038/s41598-023-42214-x (PMC10495451; doi:10.1038/s41598-023-42214-x)
Supplement: Supplementary file 1 — Supplementary Information. [file 41598_2023_42214_MOESM1_ESM.pdf]

Supplementary Material:
Material to System-Level Benchmarking of CMOS-Integrated RRAM with Ultra-fast Switching for Low Power On-Chip Learning

Minhaz Abedin[1,4], Nanbo Gong[2], Karsten Beckmann[1,3], Maximilian Liehr[1], Iqbal Saraf[4], Oscar Van der Straten[4], Takashi Ando[2], and Nathaniel Cady[1,*]

[1]SUNY Polytechnic Institute, College of Nanoscale Science and Engineering, Albany NY, 12203, USA
[2]IBM Thomas J. Watson Research Center, Yorktown Heights NY, 10598, USA
[3]NY CREATES, Albany NY, 12203, USA
[4]IBM Research, Albany NY, 12203, USA [*]cadyn@sunypoly.edu

Pulsed IV sweep for HfOx and TaOx RRAM devices:

The figure below shows the current vs voltage (IV) for set (positive voltage) and reset (negative voltage) switching.

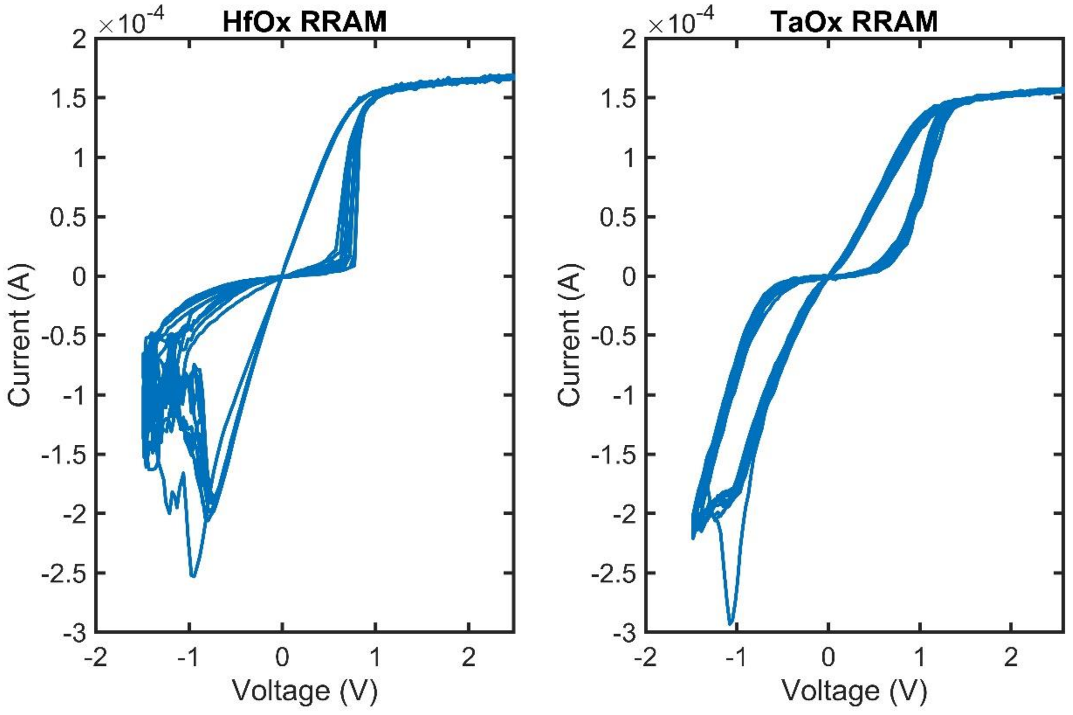

Figure: Pulsed IV data for HfOx (left) and TaOx (right) RRAM devices.

Example device thickness optimization process:

This shows with higher oxygen exchange layer (OEL) thickness (7nm to 12nm), the device forming voltage gets reduced. Furthermore, it shown in the table that keeping the same OEL thickness, lower switching layer thickness results in lower forming voltages for the device. However too thin oxide thickness would result in permanently shorted device or unfunctional device. Similar approach has been taken for HfOx device stack optimization.

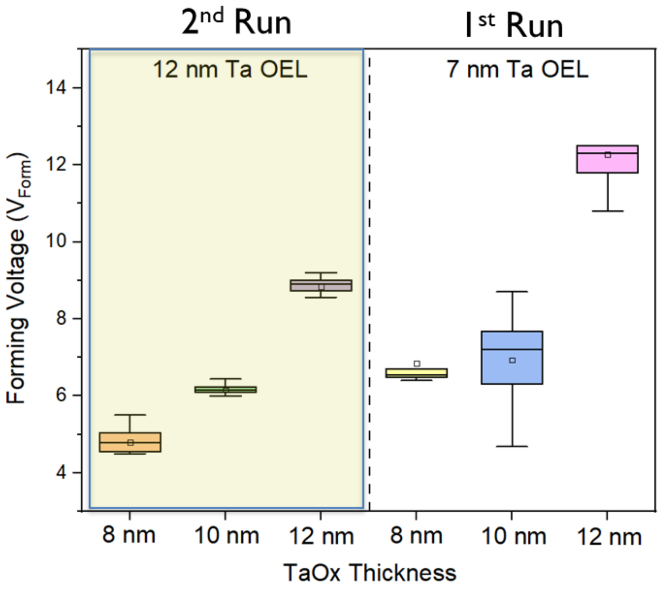

Figure: RRAM device optimization with different switching layer thickness and OEL thickness.

Table: RRAM device optimization with different switching layer thickness

CMOS 1T1R RRAM wafers	
	**Nominal Forming Voltage**
60A TaOx + 120A Ta + 40A TiN	No forming, Shorted
70A TaOx + 120A Ta + 40A TiN	Formed at ~3.3V
80A TaOx + 120A Ta + 40A TiN	Formed at ~4.1V

IV plot for the transistor integrated with RRAM.

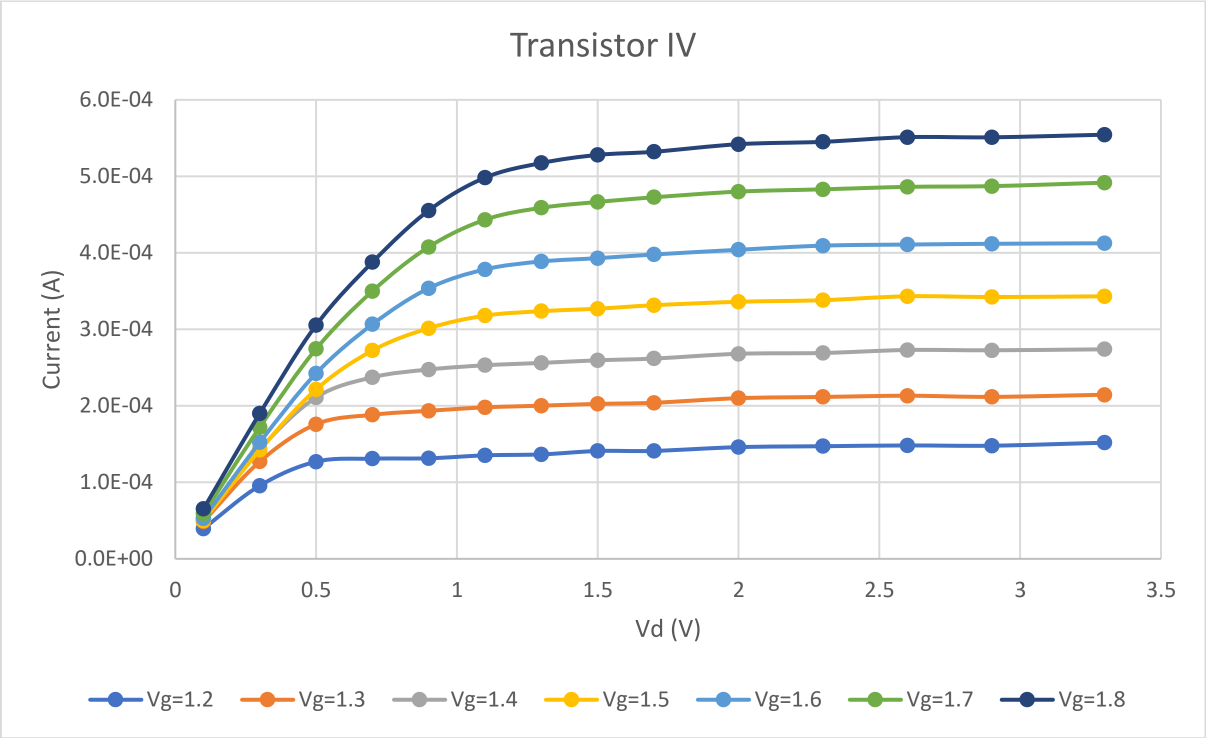

Figure: RF transistor IV sweep for different gate voltages. 1.1 V - 1.5 V used for switching doesn't fall within the linear region. This switching voltage range falls within saturation regime for the integrated transistor
